# A Systematic Review of the Diagnostic Role of BRAF V600E Testing in Thyroid Nodules with Atypia of Undetermined Significance

**DOI:** 10.7759/cureus.90292

**Published:** 2025-08-17

**Authors:** Nawfal Saleem, Mackenzie Robbins, Benjamin Kelley, Marcia Ballantyne

**Affiliations:** 1 Otolaryngology, Lake Erie College of Osteopathic Medicine, Bradenton, USA; 2 Pathology, Lake Erie College of Osteopathic Medicine, Bradenton, USA; 3 Otolaryngology - Head and Neck Surgery, Manatee Memorial Hospital, Bradenton, USA

**Keywords:** atypia of undetermined significance, bethesda 3, braf mutation, clinical decision-making, indeterminate thyroid nodules, papillary thyroid carcinoma, risk of malignancy, thyroid nodule

## Abstract

The BRAF V600E mutation is a key molecular marker for malignancy in thyroid nodules showing cytological features of atypia of undetermined significance (AUS). It is strongly associated with papillary thyroid carcinoma (PTC), improves diagnostic accuracy and clinical management stratification, and serves as a significant risk factor of malignancy (ROM) when combined with identifying aggressive features such as lymph node metastases and extra-thyroid extension. This systematic literature review examines the diagnostic role of the BRAF V600E mutation in AUS nodules, emphasizing its integration with cytological evaluation, ultrasound-based risk stratification systems (US-RSS), and cytological subclassification. Selective BRAF testing improves sensitivity and specificity, optimizing resource utilization while reducing unnecessary surgeries. Incorporation of this mutation into multigene classifiers, such as ThyroSeq v3, further supports cost-effective and personalized management strategies for indeterminate thyroid nodules. By combining molecular and imaging data, BRAF V600E testing plays a pivotal role in advancing thyroid cancer diagnosis and treatment. It enhances malignancy risk stratification and facilitates precise clinical decision-making, leading to improved patient outcomes.

## Introduction and background

Thyroid nodules are frequently encountered in clinical practice, with an estimated prevalence ranging from 19% to 68% in the general population, depending on the method of detection [1]. Among these, approximately 5-15% are classified as indeterminate based on cytological evaluation, specifically under the category of atypia of undetermined significance (AUS) within the Bethesda System for Reporting Thyroid Cytopathology (BSRTC) [2,3]. Fine-needle aspiration biopsy (FNAB) is the gold standard for initial assessment, but approximately 30% of results fall into the AUS category, necessitating further molecular testing to refine the risk of malignancy [4]. AUS, also referred to as Bethesda III, poses a diagnostic challenge due to its uncertain malignant potential, often necessitating further diagnostic evaluations to distinguish benign from malignant lesions.

Molecular markers have become pivotal in enhancing the diagnostic accuracy for indeterminate thyroid nodules. The BRAF V600E mutation, a genetic alteration involving a valine-to-glutamate substitution at position 600 of the BRAF gene, is particularly noteworthy due to its strong association with papillary thyroid carcinoma (PTC) [5]. Studies have demonstrated that the presence of the BRAF V600E mutation is highly specific for malignancy, with specificity rates nearing 100% in some cohorts [6]. According to the 2015 American Thyroid Association (ATA) guidelines, BRAF mutation testing in AUS or follicular lesion of undetermined significance (FLUS) samples demonstrates high specificity but low sensitivity for cancer detection [7]. This is corroborated by a meta-analysis indicating that BRAF mutation analysis significantly improves the diagnostic accuracy of FNAB, particularly in indeterminate cases [8]. 

Furthermore, the integration of BRAF mutation analysis with other diagnostic modalities, such as the Thyroid Imaging Reporting and Data System (TIRADS), has been shown to increase diagnostic sensitivity and accuracy [9]. TIRADS is a standardized risk stratification system used by healthcare professionals in ultrasound imaging to assess the likelihood of malignancy in thyroid nodules based on certain sonographic features. This combined approach provides a more comprehensive assessment, facilitating better clinical decision-making.

Given its diagnostic potential, the detection of the BRAF V600E mutation has been proposed as a valuable tool in risk stratification for AUS nodules. Its identification can assist clinicians in making informed decisions regarding the necessity for surgical intervention, thus balancing the risks of unnecessary surgery against the potential for undiagnosed malignancy [7].

This systematic review aims to evaluate the diagnostic utility of BRAF gene testing in the context of AUS nodules. By reviewing its sensitivity, specificity, and clinical and financial impact, we seek to clarify the role of BRAF mutation analysis in improving the risk stratification and management of patients with indeterminate thyroid nodules.

Tumorigenesis

The BRAF V600E mutation leads to the activation of the mitogen-activated protein kinase (MAPK)/extracellular signal-regulated kinase (ERK) pathway by causing constitutive kinase activity. This mutation results from a single amino acid substitution (valine to glutamic acid at position 600) in the BRAF protein, a serine-threonine kinase. This substitution mimics phosphorylation, locking BRAF in an active conformation that continuously signals downstream effectors without the need for upstream activation by RAS. RAS or “rat sarcoma” is a family of proteins that function as guanosine triphosphatases (GTPases) that play a role in cell growth, proliferation, and differentiation.

**Figure 1:**
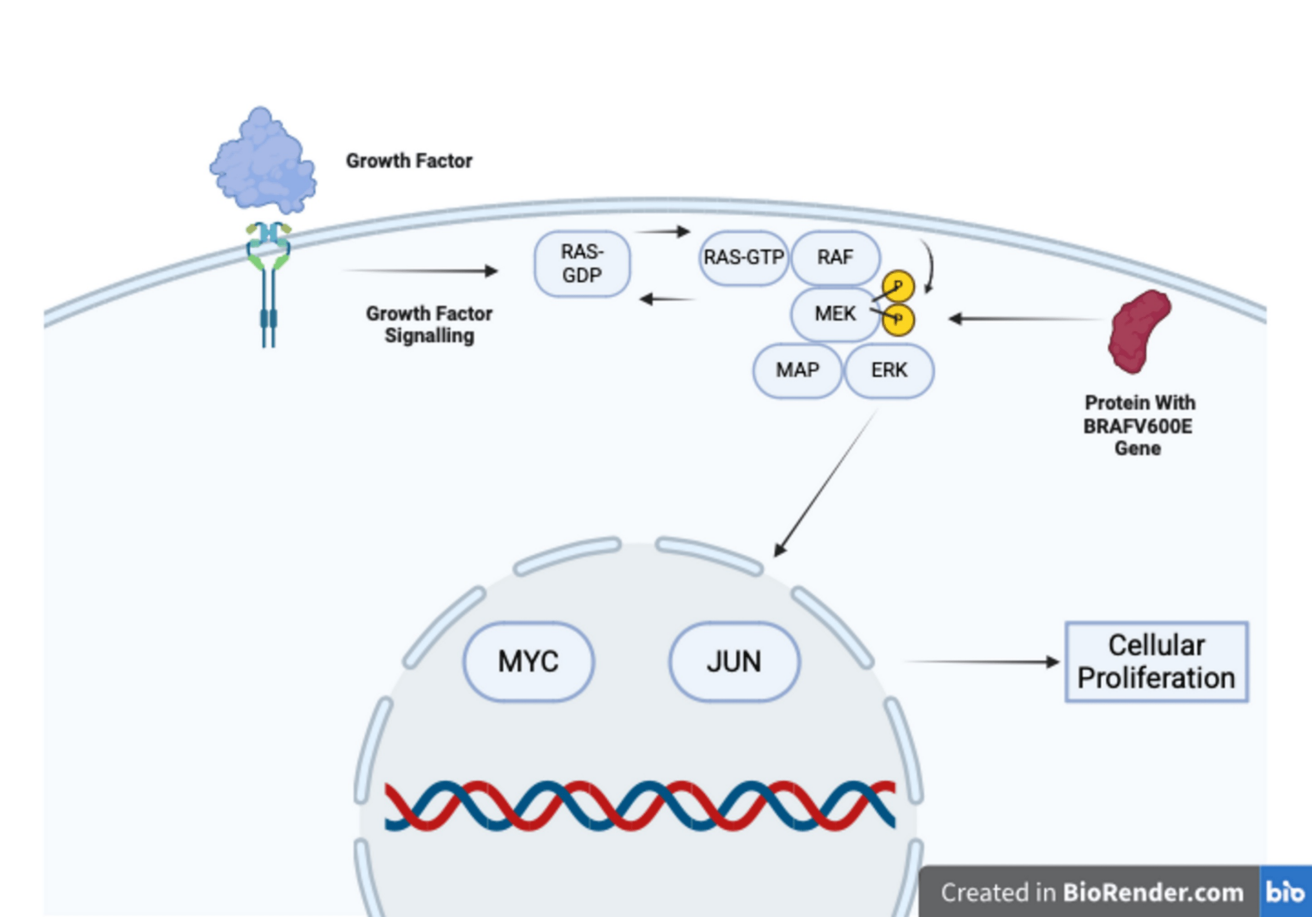
BRAF V600E and RAS/MAP pathway The illustration depicts the Ras-mitogen-activated protein kinase pathway (RAS/MAPK) signaling cascade and the impact of the BRAF V600E mutation on cellular proliferation. Growth factor binding initiates receptor activation, leading to the conversion of RAS-Guanosine diphosphate (GDP) to RAS-Guanosine triphosphate (GTP), which subsequently activates rapidly accelerated fibrosarcoma (RAF), mitogen-activated protein kinase kinase (MEK), and extracellular signal-regulated kinase (ERK). Normally, this cascade is tightly regulated. However, the BRAF V600E mutation results in constitutive activation of the pathway, bypassing upstream regulation. This leads to persistent ERK signaling, promoting the transcription of genes such as Myc and Jun, ultimately driving uncontrolled cell proliferation. The presence of the BRAF V600E mutation is clinically significant in thyroid nodules with atypia of undetermined significance (AUS) as it serves as a strong molecular marker for papillary thyroid carcinoma (PTC). Created with BioRender.com. Saleem, N. (2025) https://BioRender.com/60c5iqv

In the context of thyroid nodules with AUS, the BRAF V600E mutation is particularly significant. The mutation leads to persistent activation of the MAPK/ERK pathway, which promotes cell proliferation, differentiation, and survival. Specifically, BRAF V600E protein activates mitogen-activated protein kinase kinase (MEK), which in turn phosphorylates and activates ERK. Activated ERK translocates to the nucleus, where it phosphorylates c-Myc and c-Jun thereby regulating the expression of genes involved in cell cycle progression [10-12]. An illustration is shown in Figure 1.

The diagnostic value of detecting the BRAF V600E mutation in AUS thyroid nodules lies in its high specificity for PTC. The presence of this mutation in AUS nodules strongly suggests malignancy, aiding in the stratification of patients for appropriate surgical intervention. Studies have shown that BRAF V600E is associated with more aggressive tumor phenotypes and resistance to radioiodine therapy, further underscoring its prognostic significance [11-13].

Cytological features of atypia of undetermined significance 

AUS is a heterogeneous category within the BSRTC and accounts for approximately 5-15% of all FNAB results [2,3]. It is used when cytologic findings are neither clearly benign nor definitively suspicious for malignancy, requiring further evaluation for risk stratification.

**Figure 2:**
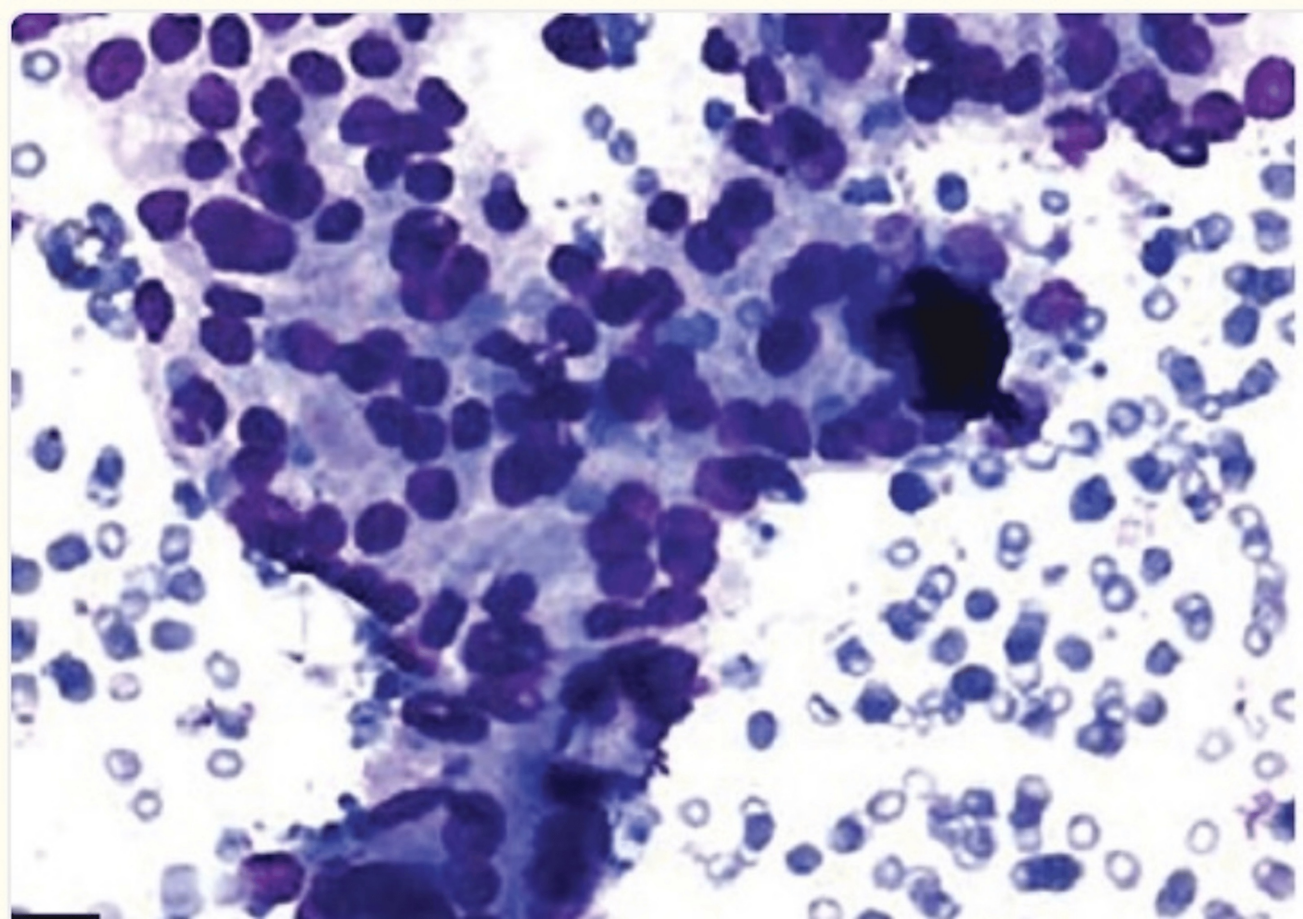
Thyroid cytopathology of Bethesda category III with microfollicles Microfollicles can be seen in the thyroid fine needle aspiration (FNA) [14].

**Figure 3:**
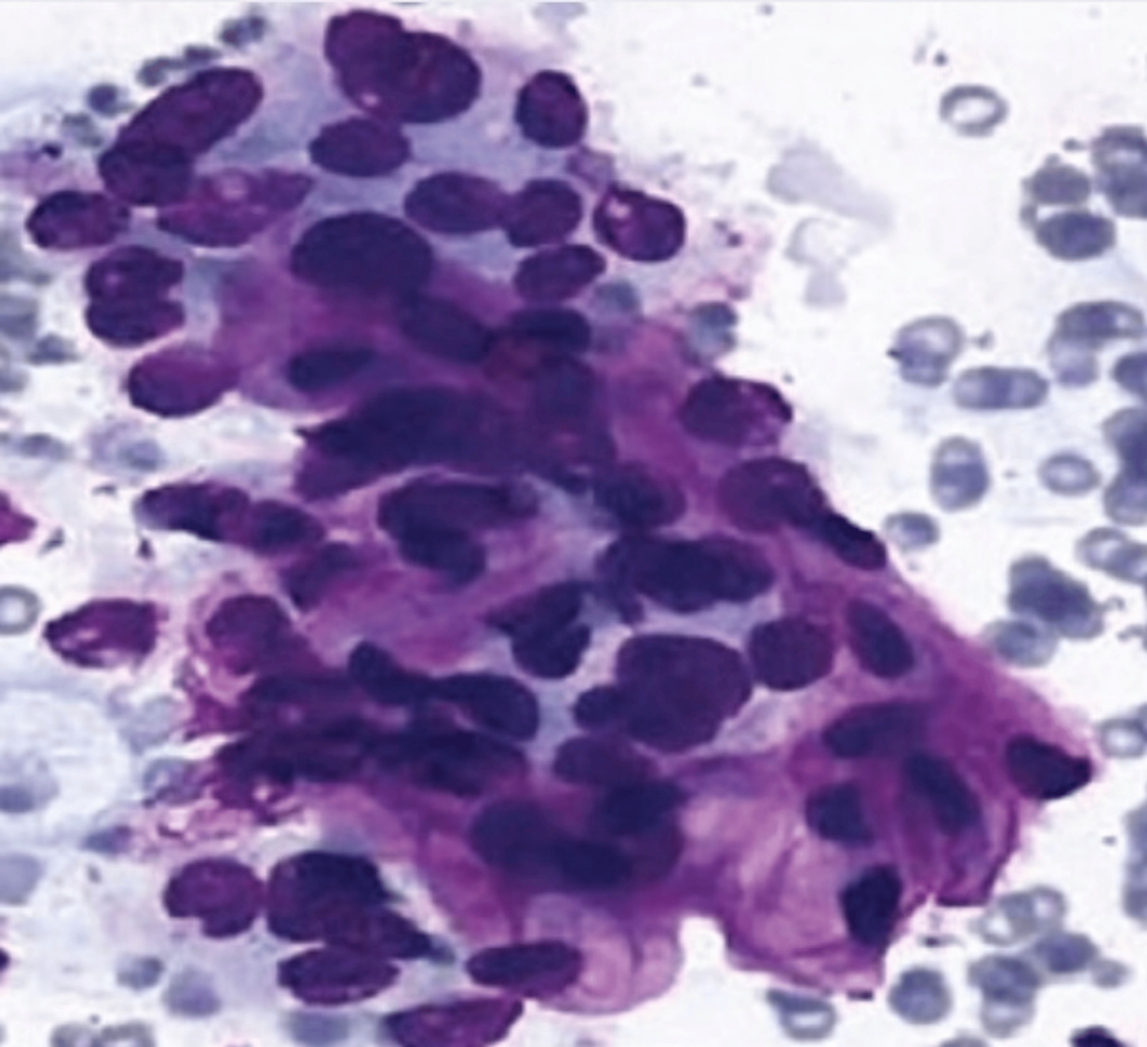
Thyroid cytopathology of Bethesda category 3 with oncocytic cells Oncocytic cells or hurthle cells show clear nuclear enlargement and a large nuclear to cytoplasm ratio [14].

AUS nodules exhibit a variety of cytological features that do not meet the criteria for follicular neoplasm, suspicious for malignancy, or malignant categories but still raise concerns for potential malignancy. The heterogeneity of this category precludes outlining all scenarios where AUS interpretation is appropriate, however the most common patterns include: (1) a prominent amount of microfollicles, which consist of a circular pattern without a true central colloid filled lumen, in an aspirate that is not abundant enough to be considered a follicular neoplasm (example shown in Figure 2);

(2) a predominance of hurthle cells (oncocytic cells), characterized by abundant eosinophilic, granular cytoplasm, and prominent, round nuclei with a large nucleolus, in a sparsely cellular aspirate; (3) a cellular sample composed of a moderate amount of hurthle cells, yet the clinical setting suggests benign hurthle cell nodule including patients with Hashimoto thyroiditis and multinodular goiter (shown in Figure 3);

(4) features suggestive of papillary carcinoma, including nuclear grooves, enlarged nuclei with pale chromatin, and alterations in nuclear contour and shape in a sample that is otherwise benign appearing; and (5) presence of atypical lymphoid infiltrate, but the degree of atypia is insufficient to be categorized as “suspicious for malignancy” [2]. 

AUS can overlap with several benign and malignant thyroid conditions, making its interpretation challenging. The differential diagnosis includes benign hyperplastic nodules, which may show mild nuclear atypia due to reactive or degenerative changes. Follicular neoplasm should be ruled out, as AUS should not demonstrate a predominant microfollicular pattern, which would otherwise place it in the follicular neoplasm category (Bethesda IV). In cases with nuclear grooves, overlapping, or clearing, suspicion may be raised for follicular variant PTC, necessitating molecular testing such as BRAF V600E analysis [2].

The malignancy rate of AUS in thyroid nodules can vary significantly based on different studies and clinical settings. The malignancy rate in AUS/FLUS settings with a repeat FNA was 38.30% and without repeat FNA was 48.11% [15]. The ATA guidelines suggest that the risk for malignancy ranges from 6% to 48% with a mean risk of 16% [7].

Since cytologic atypia alone is not sufficient for malignancy diagnosis, further evaluation through repeat FNAB, molecular testing, or ultrasound-based risk stratification is necessary [9]. AUS cases with nuclear atypia are more predictive of malignancy than those classified based on architectural atypia alone [8]. By integrating cytologic features, molecular markers, and imaging findings, clinicians can refine the risk assessment for AUS nodules and determine the most appropriate management strategy.

## Review

Methodology

**Figure 4:**
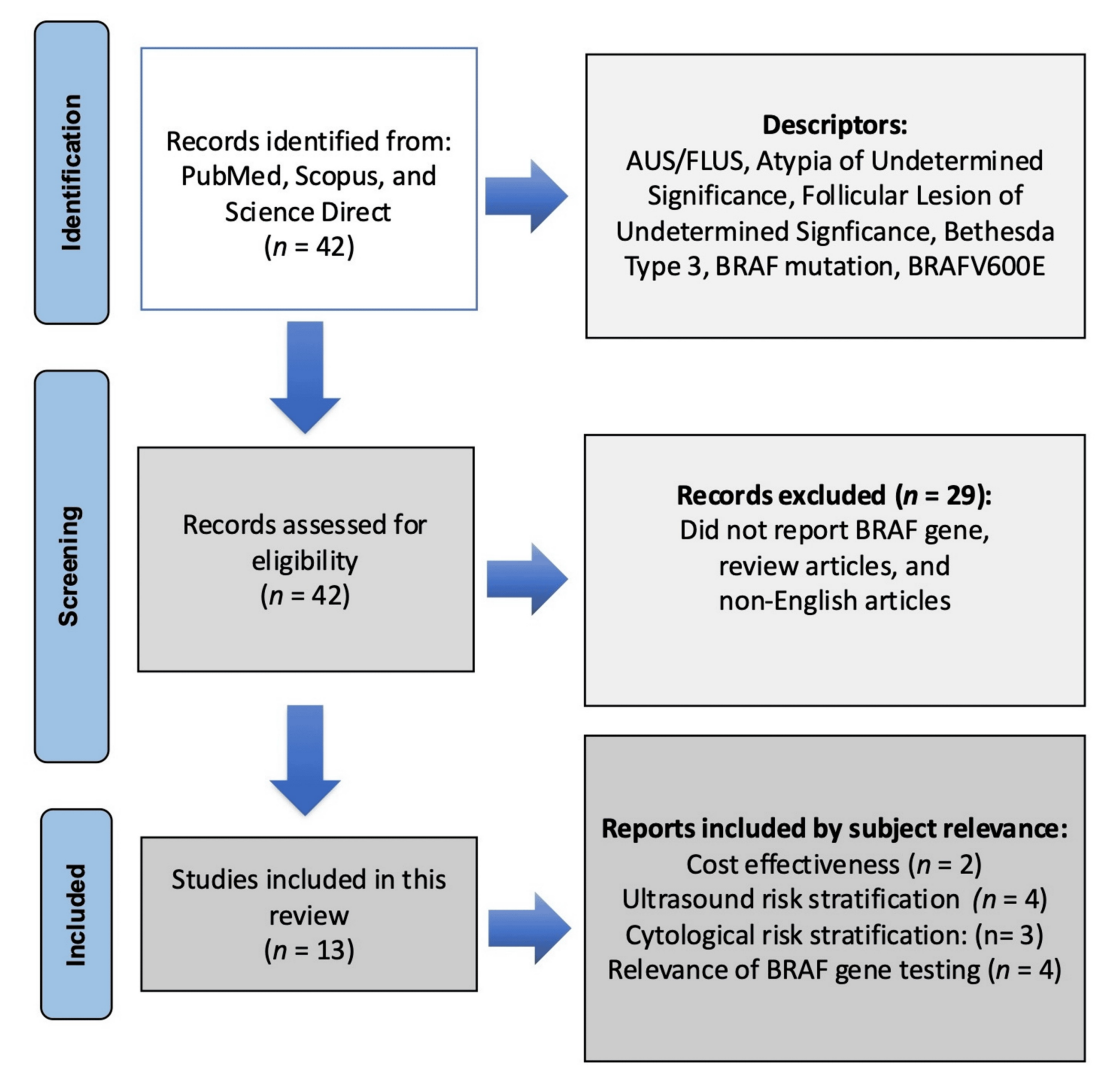
The PRISMA figure showing the steps to choose the studies for systematic review Preferred Reporting Items for Systematic Reviews and Meta-Analyses (PRISMA) [17]

This literature review was conducted in accordance with the Preferred Reporting Items for Systematic Reviews and Meta-Analyses (PRISMA) guidelines as outlined by Liberati et al. [16] and later updated by Page et al. [17]. Various scholarly databases, such as PubMed, Scopus, and ScienceDirect, were utilized as primary resources for identifying and collecting research articles relevant to this study. The literature search, carried out by all authors, spanned from September to December 2024. Keywords used in the search included, but were not limited to, AUS/FLUS, atypia of undetermined significance, follicular lesion of undetermined significance, Bethesda type 3, BRAF, V600E, and BRAF mutation. The initial screening involved reviewing titles and abstracts to identify studies that met the inclusion criteria, which was followed by a comprehensive evaluation of the full texts. The decision to include or exclude articles was guided by a detailed analysis of the study outcomes, focusing on whether the research examined the use of the BRAF gene in relation to AUS. To ensure consistency and rigor, the inclusion criteria were informed by participant, intervention comparator, and outcome (PICO) principles. Eligible studies involved individuals with AUS thyroid nodules, and the interventions under investigation were patients who received BRAF gene testing. Where applicable, comparators included alternative genetic tests or no genetic testing. The primary outcomes of interest were improvements in diagnostic accuracy, malignancy risk stratification, and cost effectiveness. Only peer-reviewed articles published in English were considered for inclusion. Articles not published in English, those lacking a clear description of the methodology, those that did not measure outcomes related to malignancy risk stratification, or focused on conditions unrelated to AUS thyroid nodules, were not included. Following the completion of the literature search, references from previous review articles were also examined to ensure that no relevant studies were overlooked. Of the initial set of 42 articles identified, 13 articles were ultimately included in this work. The literature search and screening process are illustrated in Figure 4.

Results

Table 1 summarizes the key characteristics and findings from the 13 studies included in this systematic review, each evaluating the diagnostic utility of BRAF V600E mutation testing in AUS/FLUS thyroid nodules.

**Table 1:** Study design and characteristics of articles discussing AUS/FLUS and BRAF mutation testing PTC, Papillary thyroid carcinoma; FNA, fine needle aspiration; US, ultrasound; AUS, atypia of undetermined significance; FLUS, follicular lesion of undetermined significance; US-RSS, ultrasound-based risk stratification system; ROM, risk of malignancy; C-TIRADS, Chinese thyroid imaging reporting and data system; AUS-NA, atypia of undetermined significance-nuclear atypia; FLUS, follicular lesion of undetermined significance; BRAF+, AUS/FLUS nodules with BRAF V600E mutation; TERT, Telomerase Reverse Transcriptase.

Citation	Study design	Study aim	Population/Cytology category	Key findings	Integration with other modalities	Clinical impact
Johnson et al. (2014) [18]	Pilot	Assess BRAF mutation's utility across cytologic categories in FNA samples	123 cytology specimens; Indeterminate, Suspicious, Malignant	BRAF improved diagnostic classification across cytology categories with 100% specificity for PTC	Not specified	Helped confirm malignancy
Seo et al. (2014) [19]	Retrospective	Evaluate added value of BRAF testing in nodules without suspicious ultrasound features	485 nodules in 466 patients; Suspicious for malignancy	BRAF adds value even in the absence of US suspicious features	US + BRAF	Improved diagnostic confidence in patients without suspicious US features
Decaussin-Petrucci et al. (2017) [20]	Prospective cohort	Assess diagnostic accuracy of BRAF, RAS, and TERT in indeterminate nodules	326 cases; 61 AUS; 124 follicular neoplasms; 72 suspicious; 69 malignant cases	BRAF most associated with malignancy among tested genes with 85.4% negative predictive value for AUS	Cytology + molecular	Improved sensitivity & specificity
Seo et al. (2016) [21]	Retrospective	Identify malignancy predictors in AUS nodules (clinical, US, and BRAF)	62 patients undergoing total thyroidectomy; AUS	Risk factors: suspicious US features and BRAF positivity with 100% diagnosed with PTC	US + BRAF	Improved triage of AUS cases vs “suspicious for malignancy” nodules
Zha et al. (2022) [22]	Retrospective	Evaluate tumor size and BRAF mutation as tools for risk stratification in Bethesda III nodules	1739 patients; Bethesda III	BRAF+ & tumor size > 1cm = high risk	Tumor size + BRAF	Guides precision surgery
Ma et al. (2024) [23]	Retrospective	Determine cost-effectiveness of selective BRAF testing using US-RSS	762 nodules in 551 patients; AUS/FLUS	BRAF testing targeted to medium US-RSS risk optimized cost and accuracy	US-RSS + BRAF	Cost-effective diagnosis
Kim et al. (2016) [24]	Retrospective cohort	Evaluate multimodal approach for AUS/FLUS using BRAF and US features	904 patients with initial AUS/FLUS; AUS/FLUS	BRAF V600E + US: 91% sensitivity, 84% specificity	Multimodal (Cytology + US + BRAF)	Enhanced malignancy prediction
Li et al. (2022) [25]	Retrospective	Assess diagnostic value of combining C-TIRADS with BRAF testing in AUS/FLUS	129 patients; 138 AUS/FLUS	BRAF + C-TIRADS improved sensitivity and specificity	C-TIRADS + BRAF	Enhanced malignancy prediction
Lu et al. (2023) [26]	Retrospective	Determine clinical utility of BRAF testing in Bethesda III/V nodules	12,392 patients; 5382 underwent BRAF mutation testing; Bethesda III/V	BRAF+ in Bethesda III had 98.5% malignancy rate	Cytology + BRAF	Strong surgical indicator
Steward et al. (2019) [27]	Prospective blinded multicenter	Assess performance of multigene genomic classifier (ThyroSeq v3) including BRAF	286 FNA samples; Bethesda III/IV	BRAF part of panel, sensitivity 94%, specificity 82%	Multigene panel	Avoided unnecessary surgeries
Hyeon et al. (2014) [28]	Retrospective at a single institution	Predict malignancy risk using cytological subcategorization and BRAF results	6402 FNA samples; 431 AUS; 120 FLUS; AUS/FLUS	Nuclear atypia + BRAF = highest ROM	Cytology subclassification + BRAF	Improved risk stratification
Park et al. (2014) [29]	Retrospective	Examine correlation between AUS nuclear atypia and BRAF positivity	331 AUS; AUS with nuclear atypia	Highest malignancy rate in AUS-NA; strong BRAF correlation	Cytomorphology + BRAF	Supports aggressive management
Erdogan-Durmus et al. (2021) [30]	Retrospective in tertiary center	Evaluate ROM in AUS/FLUS and assess cytological atypia patterns	1461 FNA samples; 113 AUS; AUS/FLUS	ROM of 24.6%, higher in cytologic atypia. BRAF mutation testing has low sensitivity	Cytology subclassification	BRAF mutation with cytological atypia increases malignancy prediction

The table highlights the study design, sample size, mutation prevalence, and diagnostic performance metrics such as sensitivity, specificity, and malignancy rates. Across the studies, the presence of the BRAF V600E mutation was consistently associated with a higher risk of malignancy, particularly PTC. Several studies demonstrated that combining BRAF testing with ultrasound (US)-based risk stratification or cytological subclassification further improved diagnostic accuracy, supporting its clinical value in refining risk assessment and guiding management decisions.

The role of the BRAF V600E mutation

Studies have demonstrated that the presence of the BRAF V600E mutation in AUS nodules is highly specific for malignancy, particularly PTC, with specificity reaching 100% [18,19]. Seo et al. found that the BRAF gene was able to identify malignancies even in cases when there were no suspicious findings on US [19]. This specificity makes BRAF V600E a valuable marker for distinguishing malignant nodules.

Decaussin et al. examined molecular testing for BRAF, RAS, and telomerase reverse transcriptase (TERT) mutations across 127 thyroid nodules with indeterminate cytology. They revealed 17% harbored mutations, including 8% with BRAF mutations [20]. These mutations strongly correlated with malignancy, with BRAF mutations demonstrating the highest association. This study was done in a single institution and found that integrating molecular testing with cytological evaluation improved the sensitivity and specificity of fine needle aspiration (FNA) in detecting thyroid cancer, enhancing the positive predictive value and confirming malignancy in high-risk nodules. 

A study at Dong-A University in South Korea aimed to identify clinical predictors of malignancy in thyroid nodules classified as AUS through FNA [21]. This retrospective cohort study analyzed data from 62 patients who underwent thyroid surgery between 2010 and 2013 after receiving a preoperative AUS diagnosis. The research assessed various clinical and pathological parameters, including demographic factors (age and gender), nodule characteristics (maximum size, site), ultrasonographic features, cytological results, BRAF gene mutation status, type of surgical intervention, and outcomes of repeated FNAs. Among the participants, 41 underwent total thyroidectomy, while 21 had lobectomy. Final pathological results revealed 41 malignant cases and 21 benign nodules. BRAF mutation analysis was performed in 38 patients, with 13 testing positive. All patients with the BRAF mutation were diagnosed with PTC. The study concluded that patients with thyroid nodules <1.5 cm, exhibiting two or more suspicious US features, a BRAF mutation, or multiple AUS results on repeat FNAs, warranted close monitoring or diagnostic surgery. This approach aimed to enhance the accuracy of malignancy detection while minimizing unnecessary interventions.

Zha et al. examined the role of the BRAF V600E mutation in malignancy risk stratification for thyroid nodules classified as AUS under the Bethesda III category [22]. The presence of the BRAF V600E mutation was strongly associated with aggressive features, including lymph node metastases, multifocality, and extra-thyroid extension. PTC cases with BRAF V600E mutation exhibited significantly higher odds of invasive characteristics compared to PTC with wild-type BRAF, with odds ratios of 3.01 for lymph node metastases, 3.20 for multifocality, and 5.62 for extra-thyroid extension. The study suggested that combining tumor size and BRAF mutation status can refine risk stratification within AUS/FLUS nodules. Nodules smaller than 1 cm with no mutations in the BRAF gene were considered low risk and suitable for active surveillance, while those larger than 1 cm with BRAF V600E mutation were classified as high risk, warranting diagnostic surgery.

Ultrasound-based risk stratification system (US-RSS) and molecular testing combined

The selective application of BRAF mutation analysis based on US-RSS categories has proven cost-effective. Ma et al. (2024) evaluated 762 AUS/FLUS nodules and demonstrated that targeting BRAF testing to medium-risk nodules optimized resource utilization [23]. This approach reduced unnecessary molecular testing in the setting of a positive BRAF V600E mutation. They concluded that in low-risk nodules determined by FNA and US, BRAF V600E testing should be avoided due to its low sensitivity and its inability to aid in identifying non-PTC malignancies. By stratifying nodules into low, medium, and high risk, clinicians can tailor molecular testing to cases where it has the greatest impact.

Another study looked at over 388 thyroid nodules that were found to have AUS/FLUS [24]. Key findings indicated that certain US features, such as marked hypoechogenicity, irregular or microlobulated margins, microcalcifications, and a taller-than-wide shape, were significantly associated with malignancy. When combining suspicious US features with BRAF V600E mutation analysis, the sensitivity and specificity for detecting malignancy were 91.0% and 84.0%, respectively. The study concluded that utilizing both US characteristics and BRAF V600E mutation status enhanced the diagnostic accuracy for AUS/FLUS thyroid nodules. 

Seo et al. evaluated 485 thyroid nodules with no suspicious US features, and found 12.4% of these nodules to be malignant. The study found that the BRAF mutation was present in 6.6% of the nodules. For nodules without malignant cytology, approximately 2.6% had the BRAF mutation, with most of them being classified as suspicious for malignancy on cytology. However, the mutation was not detected in the AUS nodules in this cohort. This reinforces the mutation’s diagnostic value for guiding clinical management, irrespective of US characteristics, in nodules that are suspicious for malignancy. However, its role may be limited in AUS nodules without suspicious US features [19].

Li et al. looked at over 388 thyroid nodules that were found to have AUS/FLUS [25]. The positive likelihood ratio for BRAF V600E mutation was 11.6. Key findings indicated that certain US features, such as marked hypoechogenicity, irregular or microlobulated margins, microcalcifications, and a taller-than-wide shape, were significantly associated with malignancy. For category III nodules in the ATA guidelines and Chinese Thyroid Imaging Reporting and Data Systems (C-TIRADS), the malignancy rate was found to be between 22.2 and 22.8%. C-TIRADS and BRAF V600E were both independently significant markers to test for malignancy and both were found to have similar diagnostic efficacy (p <0.05). When combining suspicious US features with BRAF V600E mutation analysis, the sensitivity and specificity for detecting malignancy were 91.0% and 84.0%, respectively. The study concluded that utilizing both US characteristics and BRAF V600E mutation status enhances the diagnostic accuracy for AUS/FLUS thyroid nodules. 

Lu et al. (2023) explored the utility of BRAF V600E genetic testing for thyroid nodules classified under the Bethesda III and V categories based on FNA [26]. The retrospective analysis revealed that BRAF V600E testing significantly benefited Bethesda III (AUS/FLUS) and V (suspicious for malignancy) nodules by enhancing diagnostic accuracy and helping guide treatment decisions. Specifically, the postoperative malignancy rate for Bethesda III nodules with a positive BRAF V600E mutation was 98.51%, suggesting its utility in determining whether surgical intervention is necessary. The study found no significant benefit of BRAF V600E testing in Bethesda I, II, IV, or VI nodules, aligning with the mutation's lower prevalence in categories I and II and high prevalence of mutations in category IV and VI. While cytology alone had high sensitivity, BRAF V600E testing showed high specificity, making their combination particularly effective in differentiating malignant from benign nodules. Testing for BRAF mutation in Bethesda III nodules can assist in classifying nodules as benign or malignant. In the setting of Bethesda V nodules, BRAF mutation testing can confirm the suspicion of malignancy, improving detection of malignancy rate from 90.21% with FNAB alone to 98.68% with added BRAF V600E genetic testing, with p=0.001. The study underscored the need for targeted use of BRAF V600E testing, and recommended it primarily for Bethesda III and V nodules to reduce unnecessary surgeries and avoid overtreatment. It emphasized the role of genetic testing in complementing cytological evaluation, aiding in clinical decision-making, and improving outcomes for patients with indeterminate thyroid nodules​.

Cost-effectiveness

Steward et al. highlighted the diagnostic value of the ThyroSeq v3 multigene genomic classifier in assessing indeterminate thyroid nodules, particularly through the inclusion of the BRAF V600E mutation [27]. BRAF V600E served as a highly specific marker of malignancy, especially for PTC, and its integration into the classifier enabled precise risk stratification. With a sensitivity of 94% and specificity of 82% for malignancy detection in Bethesda III and IV nodules, ThyroSeq v3 allowed up to 61% of patients with indeterminate cytology to avoid unnecessary diagnostic surgeries.

Its cost-effectiveness was further enhanced when BRAF V600E testing was applied selectively based on US-RSS categories [23]. Ma et al. evaluated 762 AUS nodules and found that targeting BRAF testing to medium-risk nodules optimized resource utilization and reduced unnecessary surgeries while maintaining high diagnostic accuracy. This tailored approach ensures molecular testing is applied where it provides the greatest clinical and economic benefit.

Cytological subclassification and risk stratification associated with the BRAF gene mutation

Hyeon et al. evaluated the malignancy risk of thyroid nodules classified as AUS or FLUS by focusing on the BRAF mutation analysis [28]. Subclassifying these nodules into AUS found increased risk of malignancy compared to FLUS. Out of the 147 AUS nodules, 87 were found to test positive for the BRAF mutation, of which 86 were PTC. In contrast, there was only one case of BRAF mutation out of the 120 FLUS nodules, correlating with FLUS low risk of malignancy.

Park et al. found that sub-classification of AUS into nuclear atypia and architectural atypia revealed that nuclear atypia carried a higher risk of malignancy (28.8%) compared to architectural atypia (7.1%) [29]. Nuclear atypia refers to abnormal patterns seen in the nucleus while architectural atypia refers to abnormal patterns or arrangements in the cells. About 40% of nodules with nuclear atypia revealed positive BRAF mutations, whereas only 2.8% of nodules with architectural atypia showed BRAF-positive mutations. This suggested that nuclear atypia may indicate a greater malignancy risk, supporting the need for closer monitoring or intervention in such cases. Most nodules with confirmed malignancies were ≥1 cm in size, and features such as microcalcifications and solitary nodules were associated with higher malignancy rates. In summary, AUS nodules with nuclear atypia were associated with a higher risk of malignancy and BRAF V600E mutations compared to other subset of AUS nodules. Incorporating cytological subclassification and BRAF mutation analysis enhances diagnostic accuracy and supports targeted intervention for high-risk nodules. 

Erdogan et al. examined whether cytomorphology-based subcategorization of the AUS category in thyroid FNA can better predict malignancy risk and how these subcategories correlate with the presence of the BRAF V600E mutation [30]. A total of 331 AUS cases, identified from 3589 thyroid FNAs conducted between January 2010 and December 2011, were subcategorized based on cytomorphological features: nuclear atypia (NA), microfollicle formation (MF), hurthle cell change (HC), and others (O). The overall malignancy rate for AUS cases was 23.3%. Among the subcategories, AUS-NA had the highest malignancy rate at 14.5% which the BRAF V600E mutation was most highly correlated with. These findings indicated that atypia of undetermined significance-nuclear atypia (AUS-NA) represented the highest risk for malignancy and was most strongly associated with the BRAF V600E mutation. The study concluded that combining cytomorphology-based subcategorization with BRAF V600E mutation testing provided critical insights for risk stratification and could enhance the preoperative diagnostic accuracy for AUS thyroid nodules. This approach may optimize patient management by identifying cases requiring more aggressive clinical or surgical intervention.

Discussion

The BRAF V600E mutation in thyroid nodules with AUS has significant diagnostic implications for PTC. This mutation is highly specific for PTC, with some studies showing a specificity of 100% and a positive predictive value of 100% for malignancy in such nodules. The presence of the BRAF V600E mutation in AUS nodules significantly increases the likelihood of PTC, with malignancy rates as high as 97.5% in mutation-positive nodules [30,31].

Diagnostic Significance and Risk Stratification

The BRAF V600E mutation enhances the diagnostic accuracy of FNAB for AUS nodules. Combining FNAB with BRAF V600E testing improves the detection rate of PTC, increasing the sensitivity and specificity of the diagnostic process [30,31]. This combined approach is particularly useful in stratifying the risk of malignancy in indeterminate thyroid nodules, allowing for more precise management decisions.

Integration with Cytological Evaluation and US-RSS

The integration of BRAF V600E mutation testing with cytological evaluation and US-RSS further refines the diagnostic process. US features such as taller-than-wide shape, well-defined but irregular margins, and microcalcifications are associated with higher malignancy rates in AUS nodules [32]. When combined with BRAF V600E testing, these US features can significantly enhance the predictive accuracy for PTC. Furthermore, BRAF mutations do not correlate with AUS nodules that do not have suspicious US features [19]. The combined approach effectively guides clinical decisions, potentially reducing unnecessary surgeries and improving patient management.

Cost-Effectiveness and Limitations

While BRAF V600E mutation testing is highly specific, it has limitations, including lower sensitivity compared to other molecular panels. The ATA guidelines recommend using a panel of mutations for higher sensitivity [7]. The cost-effectiveness of BRAF V600E testing is improved when combined with US findings and included in multigene classifiers (such as ThyroSeq v3), which offer a broader assessment of genetic alterations [33].

Several methodological limitations should be acknowledged as well. Although the initial search yielded 42 articles, only 13 met the inclusion criteria, reflecting a small sample size of studies to represent the evidence on the practice of BRAF mutation testing in AUS nodules. Additionally, some of the included studies had limited participant numbers, which may reduce generalizability of findings. Differences in patient populations, institutional practices, and diagnostic protocols further complicate direct comparisons and synthesis. Another limitation is the exclusion of non-English language publications and unpublished literature. This may introduce publication bias, as studies with negative findings are less likely to be published. 

## Conclusions

BRAF V600E mutation testing significantly enhances the diagnostic accuracy of FNAB for AUS nodules, particularly when integrated with cytological and US evaluations. This approach improves malignancy risk stratification and aids in clinical decision-making, although it is most effective when used as part of a comprehensive molecular testing panel.

Despite the strengths of BRAF V600E testing, it is important to acknowledge its limitations. While the mutation is highly specific for PTC, its sensitivity remains limited. As a result, a negative BRAF V600E test does not completely exclude malignancy, necessitating the continued use of complementary diagnostic tools such as repeat FNAB, hemithyroidectomy, or close clinical follow-up. Additionally, the mutation's low prevalence in follicular thyroid carcinoma and other non-PTC malignancies highlights the need for broader molecular panels in indeterminate thyroid nodules.

The integration of BRAF V600E mutation analysis with cytological evaluation and US-RSS enhances the diagnostic accuracy for AUS thyroid nodules. This approach not only improves malignancy risk assessment but also contributes to personalized patient management, ultimately leading to more informed clinical decision-making and better patient outcomes.
